# A Multiplexed Microfluidic Platform for Bone Marker Measurement: A Proof-of-Concept

**DOI:** 10.3390/mi8050133

**Published:** 2017-04-25

**Authors:** Patricia Khashayar, Ghassem Amoabediny, Bagher Larijani, Morteza Hosseini, Rik Verplancke, David Schaubroeck, Steven Van Put, Farideh Razi, Michel De Keersmaecker, Annemie Adriaens, Stefan Goemaere, Tom Fiers, Jan Vanfleteren

**Affiliations:** 1 Nanobiotechnology Department, Faculty of New Sciences & Technologies, University of Tehran, Tehran 1439957131, Iran; patricia.kh@gmail.com (P.K.); hosseini_m@ut.ac.ir (M.H.); 2 Center for Microsystems Technology, Imec and Ghent University, Gent-Zwijnaarde 9052, Belgium; rik.verplancke@ugent.be (R.V.); david.schaubroeck@ugent.be (D.S.); steven.vanput@ugent.be (S.V.P.); 3 Osteoporosis Research Center, Endocrinology and Metabolism Clinical Sciences Institute, Tehran University of Medical Sciences, Tehran 1411413137, Iran; 4 Department of Biotechnology, Faculty of Chemical Engineering, School of Engineering, University of Tehran, Tehran 1417614418, Iran; amoabedini@ut.ac.ir; 5 Nanobiotechnology Department, Research Center for New Technology in Life Sciences Engineering, University of Tehran, Tehran 1417614418, Iran; 6 Endocrinology and Metabolism Research Center, Endocrinology and Metabolism Clinical Sciences Institute, Tehran University of Medical Sciences, Tehran 1411413137, Iran; larijanib@tums.ac.ir; 7 Diabetes Research Center, Endocrinology and Metabolism Clinical Sciences Institute, Tehran University of Medical Sciences, Tehran 1411413137, Iran; Farideh.razi@gmail.com; 8 Department of Analytical Chemistry, Ghent University, Krijgslaan 281 S12, Ghent 9052, Belgium; michel.dekeersmaecker@gmail.com (M.D.K.); annemie.adriaens@ugent.be (A.A.); 9 Unit for Osteoporosis and Metabolic Bone Diseases, Ghent University Hospital, Ghent 9052, Belgium; stefan.goemaere@ugent.be (S.G.); tom.fiers@ugent.be (T.F.)

**Keywords:** biosensor, bone turnover markers, osteoporosis, gold nanoparticles, electrochemistry

## Abstract

In this work, we report a microfluidic platform that can be easily translated into a biomarker diagnostic. This platform integrates microfluidic technology with electrochemical sensing and embodies a reaction/detection chamber to measure serum levels of different biomarkers. Microfabricated Au electrodes encased in a microfluidic chamber are functionalized to immobilize the antibodies, which can selectively capture the corresponding antigen. An oxidative peak is obtained using the chronoamperometry technique at room temperature. The magnitude of the response current varies linearly with the logarithmic concentration of the relative biomarker and, thus, is used to quantify the concentration of the relative biomarker in serum samples. We demonstrated the implementation, feasibility and specificity of this platform (Osteokit) in assaying serum levels of bone turnover markers (BTMs) using osteocalcin (limits of detection (LOD) = 1.94 ng/mL) and collagen type 1 cross-linked C-telopeptide (CTX) (LOD = 1.39 pg/mL). To our knowledge, this is the first such device fabricated to measure BTMs. Our results also showed that the sensitivity of Osteokit is comparable with the current states of art, electrochemiluminescence (ECLIA).

## 1. Introduction

Current trends towards theranostics and the provision of personalized diagnostic therapy tailored to an individual has emphasized the need for inexpensive point-of-care (PoC) devices capable of performing rapid analysis, with small volumes of sample, minimum number of assay steps, and no need for highly-skilled personnel for routine checkups and patient screening [1,2]. This is of greater importance in remote and resource-limited settings, such as developing countries, where access to central laboratories is limited and, thus, on-site diagnosis can lead to improved health status and quality of life [3].

This is while enzyme-linked immunosorbent assay (ELISA), electrochemiluminescence immunoassay (ECLIA), and other prevailing laboratory techniques in the current state of the art for biomarker quantification do not lend themselves well to miniaturization for application at the point-of-care [4]. Thus, performing rapid measurements to monitor several biochemical parameters at the same time to reach an accurate medical decision still remains a largely unmet challenge.

In this context, multiplexed detection of different biomarkers using microfluidic systems has attracted considerable interest [5,6]. This is mainly due to the fact that such systems provide a promising method to miniaturize immunoassays, which have played an important role in the diagnosis of different diseases in the past 50 years. Such devices also provide added benefits, such as portability, increased reliability, improved sensitivity, decreased analysis time, minimal reagent consumption, and parallel processing [7,8]. In other words, the high surface-to-volume ratio of the microfluidic channels facilitates faster and more sensitive reactions, which is of great importance in meeting the major demands for the on-site detection of various analytes [9,10].

Among different techniques used to generate signals in these platforms, electrochemical methods have shown the most suitable results because of their simple instrumentation, easy signal quantification, rapid response, low cost, portability, reliability, good sensitivity, and excellent selectivity [11,12]. The sensitivity of biosensor platforms depend on various parameters, such as the microfluidic flow, as well as the specific surface area, conductivity, and charge transfer properties of the sensing platform [9]. In this regard, functionalized gold nanoparticles (AuNPs) have shown promising results in improving the performance of the system through facilitating the electron exchange between the antibody complex and the electrode [13].

Despite the recent improvements in the development of multiplexed microfluidic-based systems, especially in personalized cancer diagnostics, a clear challenge still resides in the integration and operation of such microfluidic platforms for the measurement of different biomarkers in clinical practice [14,15,16]. As such, a few attempts have been made in the past for the development of new screening assay methods for bone turnover markers (BTMs); yet none have succeeded to fully satisfy the criteria needed for their application in clinical practice [17]. BTMs are important determinants of bone strength as they demonstrate the bone-remodeling rate by assessing bone resorption and formation [18,19]. Monitoring the efficacy of bone-active drugs is currently the most promising clinical application for BTMs since, by using this technique, the changes in BTM levels in response to therapeutic interventions would be measurable in a shorter time interval (as early as 4–12 weeks) [20]. Moreover, the pre-treatment levels are also useful in identifying the patients who will most benefit from the treatment. This is while “the routine use of biochemical markers of bone turnover in clinical practice is not generally recommended, as these tests vary in sensitivity and specificity and their appropriate role in patient management is not yet known [21].” Thus, measuring bone mineral density (BMD) using dual energy X-ray absorptiometry (DXA) is currently considered as the gold standard for osteoporosis diagnosis despite its proven setbacks [22,23].

In the present work, we report the design and fabrication of a sensitive microfluidic platform integrated with electrochemical sensing that can easily be translated into a protein biomarker diagnostic. To our knowledge, this is the first microfluidic-based device designed as a step toward developing a PoC system for the measurement of several BTMs.

## 2. Results

### 2.1. Osteokit Characterization

Having a well-dispersed layer of AuNPs with a uniform size distribution is the first and a critical step in surface modification. The deposition time and scan rate are, therefore, properly controlled to assure sufficient deposition and uniform distribution of AuNPs [24]. The electrochemical behavior of the stepwise fabrication of the electrochemical sensor chip was studied using cyclic voltammetry (CV). Compared with conventional electrodes, the as-prepared AuNPs-based electrode-on-chip showed a 7.5-fold larger current response [24].

As mentioned in our previous article, the adsorption of the antibodies onto the working electrode was associated with a significant reduction of the conductive area of the working electrode, as well as a considerable increase in its electric resistance, resulting in a significant decrease in the magnitude of the electrochemical response current [25]. This suggests successful immobilization of antibodies on the electrode.

Later, the presence of antigens on the working electrode resulted in an increase in the double-layer capacitance and, hence, reduced electrochemical behavior of the test solution proportional to the concentration of the antigen. This was used as the basis for the determination of antigen concentration and plotting the calibration curve.

### 2.2. Calibration of the Osteokit

The electrochemical response of the Osteokit was studied as a function of different concentrations of osteocalcin (Oc) and collagen type 1 cross-linked C-telopeptide (CTX) using chronoamperometry technique under identical experimental conditions (0.1 mM potassium ferricyanide [K_3_Fe(CN)_6_] as a redox probe, at a potential of 0.65 V for 15 s), respectively. It is noted that the sample consumption of this prototype chip was only 100 μL, which is less than traditional methods (10 mL).

**Figure 1:**
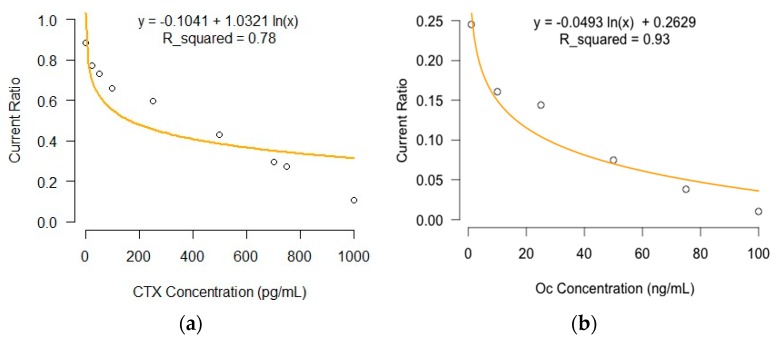
Calibration curve plotted based on the electrochemical response studies of the Osteokit as a function of (**a**) CTX concentration (1–1000 pg/mL); and (**b**) Oc concentration (1–100 ng/mL).

The magnitude of response current ratio decreased with increasing antigen concentrations; this is due to the formation of immune complexes between the antibody and antigen, resulting in a hindrance in electron charge transfer rate at the electrode–electrolyte interface (Figure 1). The magnitude of current ratio in the Osteokit with a CTX immunoelectrode was linearly dependent on the logarithmic CTX concentration with an *R*^2^ of 0.78. The correlation coefficient was 0.99 and the limits of detection (LOD) and limits of quantification (LOQ) were calculated to be 1.39 pg/mL and 4.22 pg/mL, respectively.

Similarly, the Osteokit with an Oc immunoelectrode exhibited a near-linear relationship between the current ratio and logarithmic Oc concentration from 1 to 100 ng/mL (*R*^2^ = 0.93) (Figure 1). The LOD was experimentally found to be 1.94 ng/mL. The system had a LOQ of 5.89 ng/mL with a correlation coefficient of 0.96.

The Osteokit demonstrated a large dynamic range, which was on the order of the physiological concentration of the studied antigens in the serum (reference range of Oc: 9–42 ng/mL; CTX: 25–1008 pg/mL). Notably, the titration curve revealed an apparent sub-linearity at the low end of the dynamic range for both analytes, which could be attributed to the translation of the actual number of antigens captured by the AuNPs.

### 2.3. Selectivity and Cross-Reactivity

**Figure 2:**
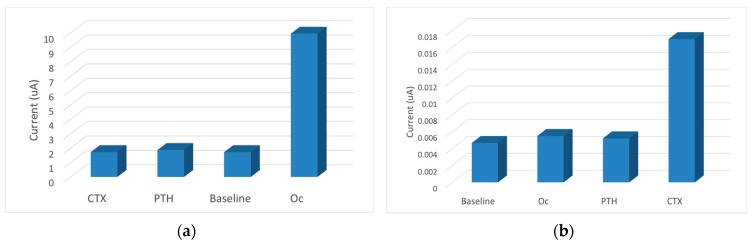
(**a**) Electrochemical response measured for the Oc sensor incubated with CTX (100 ng/mL), PTH (100 ng/mL), and Oc (100 ng/mL); and (**b**) the response measured for CTX sensor incubated with the Oc (100 pg/mL), PTH (100 pg/mL), and CTX (100 pg/mL).

In order to check the selectivity and potential cross-reactivity, the measurements were performed for a constant concentration of the opposite antigen along with Parathyroid hormone (PTH). The Osteokit showed a minimum change from the baseline value (3%–5%) with respect to Oc (100 pg/mL) and PTH (100 pg/mL) as interferents on the CTX immunoelectrode (Figure 2). Similarly there was no remarkable response when the Oc immunoelectrode was exposed to CTX (100 ng/mL) and PTH (100 ng/mL) (minimum change of 3%–5% from baseline). These results show that the Osteokit is selective for both CTX and Oc and no crosstalk occurs between the channels.

### 2.4. Stability and Repeatability

The results showed a remarkable reproducibility of the results, tested by the application of the same concentration of either antigen on different platforms (relative standard deviation (RSD) lower than 8%). The sensing interface was used ten times without sacrificing its detection efficiency. When the sensing surfaces were regenerated for more tests, the detection of either antigen was not as sensitive as before. This might be the result of the immobilized antibody losing its activity after being used repeatedly.

To investigate the stability of the chips, the sensing interfaces were stored at room temperature for a week. Compared with the relatively fresh sensing interfaces, the signal of the conserved ones only decreased by 8.1% for the Oc immunoelectrode and 8.8% for the CTX immunoelectrode. The small decrease may be ascribed to the partial degradation of the immobilized antibody.

### 2.5. Real Serum Measurements

To compare the sensitivity of our platform with the current gold standard methods, real samples (human serum) previously tested with ECLIA were used as the analyte (Oc samples were obtained from the Endocrinology and Metabolism Research Institute (EMRI) laboratory and CTX samples were obtained from Universitair ziekenhuis (UZ) Hospital). The serum of ten patients whose Oc and CTX levels were recently measured using ECLIA was used. Using the trend function and based on the calibration curve, the concentration of Oc and CTX in the tested samples were calculated according to the obtained current. The results were then compared. According to the results, the coefficients of variation for ECLIA and Osteokit were calculated to be 4.6% and 3.7% for Oc, and 6.4% and 7.7% for CTX.

**Figure 3:**
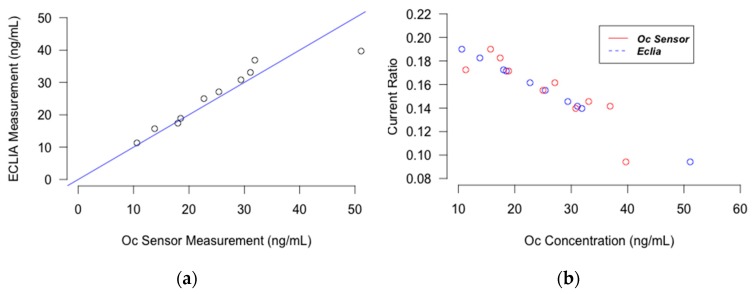
Plot displaying (**a**) the correlation between Osteokit and ECLIA results for Oc measurement (**b**) chronoamperometric response for Oc measurement using Osteokit and ECLIA.

**Figure 4:**
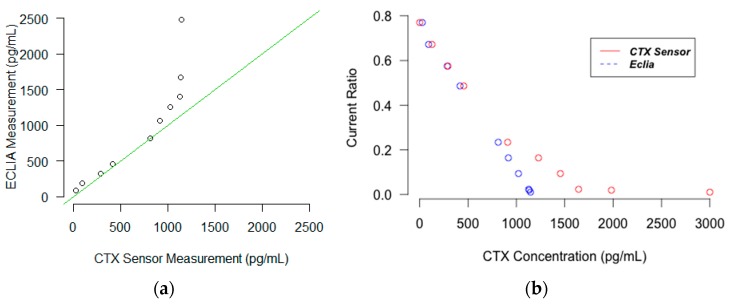
Plot displaying (**a**) the correlation between Osteokit and ECLIA results for CTX measurement (**b**) chronoamperometric response for CTX measurement using Osteokit and ECLIA.

The correlation between the results of the sensor and that of ECLIA was investigated and the results are depicted as the correlation and Bland and Altman plots in Figure 3 and Figure 4. The *R*^2^ of the trend line in the correlation plot was 0.85 for Oc and 0.87 for CTX. This indicates that the Osteokit has an acceptable correlation with ECLIA in measuring both Oc and CTX. However, the results are more acceptable for lower serum levels of CTX. This could be explained due to the fact that the calibration curve was plotted for CTX concentrations between 1 and 1000 pg/mL and the ratio for higher concentrations required for the comparison was extrapolated. The sub-linearity of the curve at the higher concentrations also aggravated the accuracy concerns.

## 3. Discussion

The improvement of detection sensitivity and analysis time has been a key motivation for the development of analytical microsystems [26,27]. Size reduction lowers the consumed reagent and sample volume and, thus, could help with portability of the device. This, however, may compromise the detection limit of the device, especially for low flow rates [28]. This is while the maximum allowed pressure for reliable operation and integrity of the microfluidic device limits the flow rate [29].

In this perspective, we developed an electrochemical biosensor for BTM measurement in a microfluidic chip architecture for identifying individuals at risk of fracture or monitoring the treatment process in osteoporotic patients. In order to improve sensitivity and specificity, we addressed the following parameters:

(1) Successful and functional immobilization of the antibodies on sensor surfaces to perform reagentless detection;
(2) Amplification of reaction signals.

It is known that adsorption of proteins onto bulk metal surfaces leads to their denaturation and loss of bioactivity [30]. Unstable immobilization of antibodies on the electrode surface, as well as nonspecific binding, are the main factors that determine the detection limit for an immunoassay [31]. Our previous work attempted to address this issue [25]. In the presented work, modification of the gold electrode with AuNPs provided a suitable environment for stable immobilization of BTMs, while keeping their bioactivity in a microreactor environment and, thus, supporting high-quality and low-background electrochemical sensing. The biomarkers were immobilized on the treated surface through covalent binding using cross-linkers. Immobilization of proteins based on the formation of covalent bonds is among the most widely used techniques, mainly because of the stable nature of the resulted complex [32,33]. The efficiency of this technique was confirmed, as mentioned earlier [25].

Moreover, the use of high specific antibodies as a bioaffinity sensing interface not only improved the selectivity, but also allowed detection with significantly low amounts of the sample.

(3) Flawless flow of the solutions in the microchannels during the preparation steps and electrochemical process.

In this work, the analyte flowed in and out of the chamber, achieved through capillary motion and pressure-driven flow, generated by a syringe. The flow behavior mainly depended on the inlet flow rate and, thus, the liquid was introduced into the system with a constant volumetric flow rate [34]. Very low and high flow rates were observed to be associated with gas bubble introduction in the channels. Moreover, the burst test results showed that the inlet flow rates higher than 5000 µL/min may fail either at the point of attachment of the interconnect, or at the interface between the two bonded parts [35].

Liquid movement was also controlled by different fluidic resistance within the parallel channel network. During the experiment, the pressurized solution mainly flowed into the reaction chamber as the fluidic resistance of other channels, which were filled with air at this point, was higher than that of the reaction chamber. The adopted design was also accountable for reduced back flow and improved the movement of the solutions without gas bubbles being trapped in the channels and affecting the detection signal. Moreover, a wash step was performed at the beginning to ensure the removal of the remaining air in the interconnects, channels, and chambers. This is important because when hydraulic pressure is applied, gas bubbles remaining in the interconnection holes can be trapped in the reaction chamber and cause significant problems in the detection process by disturbing the fluid flow or drying out the electrodes [36].

(4) Reduced reagent adsorption in channel walls and the absence of cross-talk due to the diffusion of electroactive products from one electrode to the neighboring one.

The use of the microchannels provided controlled functionalization of the sensing platform, while preventing possible contamination and crosstalk.

One of the main purposes of the fabrication of microfluidic devices with feature size in the micrometer range is to reduce the quantity of the needed sample. In this regard, non-specific adsorption of proteins to channel walls should be prohibited as it may cause an unpredictable reduction in the concentration of the assay component and slow its transport rate. As a result, controlled protein adsorption characteristics of the material used in the microchannel structure is of great importance. These materials should also provide proper channel enclosure without deforming small features or clogging the channels during the bonding process [37,38]. To overcome these concerns, we used tape both as a glue to bond layers and as a channel structure [35]. The double-sided tape provided a uniform thickness for keeping the bonding yield high, preventing leakage, delamination, and channel rupture under applied pressure. It also enabled the design of low feature size patterns.

On the other hand, the combination of the adhesive tape and the milled holder offered a means to rapidly fabricate prototype devices and, thereafter, the refinement of the design to meet the required performance was easier. In other words, using the tape, channel fabrication became rapid and simple. Moreover, the modular design of the chip allowed us to easily modify the microfluidic manifold and the electrochemical-sensing components separately. Thus, modification was no longer an outlandish task.

## 4. Materials and Methods

### 4.1. Chemicals

L-glutathione reduced (GSH), chloroauric acid (HAuCl_4_·3H_2_O), b-1-ethyl-3-(3-dimethylamonipropyl) carbodiimide (EDC), sulfo-*N*-hydroxysuccinimide (s-NHS), bovine serum albumin (BSA), phosphate buffered saline (PBS), PBS-Tween 20, and potassium hexacyanoferrate (III) (K_3_[Fe(CN)_6_]) were purchased from Sigma-Aldrich (Diegem, Belgium). PBS solution (10 mM, NaCl 0.138 M, KCl 0.0027 M, pH 7.4), and PBS-Tween 20 were prepared by dissolving one package in 1000 mL of de-ionized (DI) water. PBS was used to prepare the antibody (10 µg/mL) and antigen solutions. All other solvents and chemicals were of analytical grade.

For functionalization of the electrodes, monoclonal osteocalcin antibody (ab133612) and full-length osteocalcin protein (ab152231) was obtained from Abcam Co. (Cambridge, UK). Anti-collagen type I antibody (MAB1340) and human collagen type I (CC050) were obtained from Merck Chemicals (Overijse, Belgium).

Double-coated polyester diagnostic tape 9965 (3M Medical Specialties, St. Paul, MN, USA) was used. It consisted of a 0.05 mm opaque white polyester film coated on both sides with a 0.02 mm neutral pressure sensitive acrylate adhesive and supplied between two clear, 0.05 mm silicone-coated polyester release liners.

### 4.2. Apparatus

All electrochemical experiments were performed using a computer-controlled Autolab PGSTAT101 (Metrohm, Antwerpen, The Netherlands). Cyclic voltammetry (CV) and chronoamperometry were utilized to characterize the stepwise fabrication of the device and the antigen quantification. An electroactive substance, 0.1 mM K_3_[Fe(CN)_6_] containing 0.01 M NaCl, was used as the electrolyte solution for electrochemical readings. All electrochemical measurements were performed in a standard three-electrode format at room temperature.

In order to check the accuracy of the system, ECLIA immunoassay (Elecsys 2010 autoanalyzer, Roche Diagnostics GmbH, Mannheim, Germany) was used to measure serum levels of Oc (intra- and interassay coefficients of variation 1.2%–4.0% and 1.7%–6.5%, respectively) and CTX (intermediate precision CV of <20%).

### 4.3. System Design and Fabrication

#### 4.3.1. Design and Fabrication of Sensor Chip

**Figure 5:**
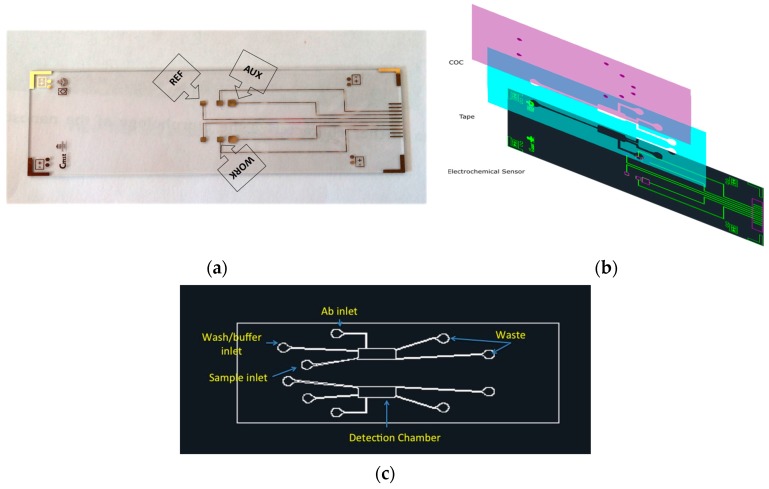
Diagram of the microsystem device: (**a**) schematic of the electrochemical sensor chip; (**b**) CAD design of each layer; and (**c**) the structure of the microfluidic manifold.

The designed electrochemical chip consisted of six gold electrodes arranged in a 2 × 3 electrode array on glass. The glass was 25.5 mm × 85.5 mm, leaving a 25.5 mm × 10 mm uncovered area for the contact pads to be connected to the reader using a printed circuit board (PCB). Each gold working electrode (1.5 mm × 1.5 mm) was placed between one gold pseudo-reference (1.4 mm × 1.4 mm) and one gold counter electrode (2.5 mm × 2.5 mm), as depicted in Figure 5a. The gold electrodes were fabricated using standard photolithographic methods. This method allowed the preparation of several dozens of electrodes in a single run. The detailed explanation of the process is mentioned elsewhere [24].

Briefly, 50 nanometers of titanium, used as a glass/metal adhesion promoter was sputtered followed by a 100 nm thick gold layer. The substrate was then fully coated with the photoresist and UV patterned to reveal electrode areas and their corresponding connections. After wet etching, the remaining photoresist was removed with acetone/isopropyl alcohol (IPA). Since the connection lines between the electrodes and their connection points have to cross the fluidic channels, insulation of the chip was required. Thus, a SU8-negative photoresist was applied to the whole surface of the chip and was only partially removed at the electrode areas. This ensured that only electrodes would be later exposed to the sample fluids. A Ti promoter was used to improve SU-8 attachment to the glass.

#### 4.3.2. Design and Fabrication of the Microfluidic Manifold

An exploded view of the three-layer microfluidic manifold with the sensor chip is presented in Figure 5b. In this regard, the sensor chip (gold electrodes on the glass) acted as the bottom layer. The middle layer (tape) defined the reaction chamber and channels. The topmost layer provided the cover to the microchannels and the inlet/outlet (I/O) for sample/buffer introduction and waste, respectively. Cyclic olefin copolymer (COC), Topas 8007, was used as the top layer for several reasons: drilling the I/O holes was much easier in COC compared to glass.

In order to develop an operational electrochemical diagnostic prototype, the microfluidic platform consisted of the microchannels and a chamber necessary to expose electrodes in the electrochemical sensor chip to the sample, surface modification solution, and detection probe (Figure 5c). The mask for fabrication of the microfluidic manifold was designed using AutoCAD software (Autodesk, San Rafael, CA, USA).

For simplicity, the microfluidic channels were realized using a double-sided medical-grade tape (3M) of 86 μm thickness, which was previously laser machined to generate microchannel structures [35]. The tapes were patterned using a focused CO_2_ laser beam (9.6 μm CO_2_, mask 1000 μm (spot size 100 μm), 1.2 mm/s (seven pulses), 100 mW). The patterned tape was also used to integrate the microfluidic manifold with the glass sensor chip and aligned using a manual mask aligner (SET MG1410, SET Corporation, Saint Jeoire, France).

The reaction chamber provided the space for the incubation of sample on the electrode surface. The 2.9 × 8.9 mm capture/reaction chamber was 86 μm deep, equal to the thickness of the tape (volume = 2.2 µL), to maximize the number of interactions between the agents needed to functionalize the surface, as well as that between target analytes and the functionalized surface.

#### 4.3.3. Design and Fabrication of the Holder

As mentioned in our previous article, in order to expose the microfluidic chip to external fluidics and electrical connections, a 65 mm × 126 mm custom-made holder was designed and fabricated in-house by micro-milling in a 10 mm poly(methyl methacrylate) (PMMA) sheet [35]. The docking station, used to house the chip, functioned as a base with female ports fully integrated into the device to overcome possible chemical contamination by glue used at the fluid access holes [39]. The use of an interconnect, therefore, not only promised ease of fabrication and an absence of contamination, but also made the process reproducible and reliable. These interconnects were chosen as their small footprint allowed a high density of fluidic I/O ports and minimal dead volume.

### 4.4. Sensor Chip Preparation

#### 4.4.1. Pretreatment of Working Electrodes

In order to coat the working electrode with a gold nanoparticle layer that would allow sufficient immobilization of Abs on the surface in later steps, the CV-electrodeposition technique (scanning the potential from 0.0 V to 1.3 V at a scan rate 0.1 V/s for 100 scans in an aqueous solution of 1.0 mM HAuCl_4_ and 0.5 M H_2_SO_4_ in the presence of 0.1 mM NaCl) was used. This process is explained in detail elsewhere [24].

#### 4.4.2. Surface Modification of Sensing Interfaces

The sensing chip was then bonded with the microfluidic chip, making the device (Osteokit) ready to use. In subsequent steps, microfluidic condition was maintained in the microchannels and chambers. The sequence of operation, which led to the gold surface being subsequently modified with capturing antibodies that bound specifically to the analyte molecules (antigens), was performed according to the method previously described by the same group [25].

Briefly, the electrodes were first activated by injecting an aqueous solution of 10 mM GSH via the loading channel to the reaction chamber and held for incubation for 1 h. Then, the antibody complex was introduced until the chamber was filled with the sample and incubated for 2 h (static condition). The antibody complex was prepared by incubating the antibodies with cross-linkers (100 mM s-NHS for osteocalcin, and 400 mM EDC/100 mM s-NHS for CTX) for 2.5 h at room temperature. The cross-linkers provided covalent attractions between the constituents, facilitating the bonding of the primary amine groups (–NH_2_) of the antibody to the carboxylic group of GSH to form stable amide bonds. The active parts of the device were washed between each step with DI to remove unbound fractions. BSA solution (0.2% (*w*/*v*)) in PBS buffer was then pumped into the microchannel to immerse the treated electrode for one hour and block unreacted active functional groups. The assembly was kept at room temperature during all steps and finally rinsed with PBS and then with PBS-Tween 20 thoroughly to remove unbound analytes and other non-specific entities from the electrode surface.

#### 4.4.3. Immunosensing Strategy

To investigate the applicability of the Osteokit for osteoporosis care, we performed an electrochemical immunoassay using the fabricated device to measure serum levels of certain BTMs. All experiments were performed within a day of the chip fabrication to obtain a good sensitivity because the immobilized antibody (Ab) might be degenerated over time.

For this purpose, different concentrations of Osteocalcin and CTX were applied. The antibody-antigen reaction provided a redox current, which was interpreted by the chronoamperometry. A high-quality signal was obtained at +0.65 V, thus, this potential was taken as the best value for BTM determination. In this regard, sample solutions with different concentrations of each antigen were loaded into the corresponding chamber where it reacted with the Ab-functionalized electrode-on-a-chip. The rate of analyte captured by surface-bound antibodies can be very slow as the flow in microfluidic devices is nearly always laminar and mixing occurs only through diffusion. Differential Pulse Voltammetry (DPV) studies revealed that the magnitude of the current response decreased over time due to the increased binding between antibody and antigen, causing electron transfer resistance [25]. However, no further decrease in current was observed after 5 min, indicating saturated binding of the immunoreaction. Thus, the optimum detection time was established at 5 min.

Then, PBS solutions was flushed through the channel twice to ensure complete washing and removal of any unbonded molecules. The detection solution, Fe(CN)_6_^3−/4−^, was then introduced to fill the reaction chamber for electrochemical measurements. After the measurement, a relatively large amount of buffer solution was again injected to wash the analyte sample out and fill the reaction channel. The increasing concentrations of samples were injected sequentially onto sensing interfaces that already had samples on them. However, because of the remarkable repeatability of the Au working electrode-on-a-chip, the sensing interfaces were still functional.

The calibration curves were generated based on the average value of measurement conducted in triplet sets of certain amounts of antigens incubated on the sensing interface. It should be noted that the interface was exposed to different amounts of antigen from the lowest to the highest concentrations during this process. The limits of detection (LOD) and the limits of quantification (LOQ) were then calculated [40].

## 5. Conclusions

The proposed disposable cartridge chip consisted of two microfluidic systems. The electrodes in the reaction chamber of each of these systems was modified to measure a BTM (one Oc and one CTX) in a single run. The total assay time for this system was about 10 min (loading of antigen, incubation time, flushing with PBS, and testing), which is shorter than the time needed for ECLIA. The comparison of the results to the conventional method also revealed a high compatibility of results and, thus, promising potential for clinical applications of the Osteokit. The main objective was to develop the first such device to be used in osteoporosis care with enhanced sensitivity and reduced analysis time.

It could be concluded that the whole platform is simple in design, inexpensive, and easy to fabricate and, at the same time, offers accurate measurements. In other words, the use of microfluidic components along with the employment of the electrochemical detection, suggests that the proposed device can be operated as a handheld device in the future.

Although initial efforts were focused on the development of a chip to assay two major BTMs, this platform can be adapted to different types of proteomic assays. More specifically, this work serves as the basis for a clinical diagnostic that can be used by healthcare providers to monitor treatment efficacies. In other words, our contribution proposes a simple and rapid, yet powerful, approach for prototype microfluidics and sensor assembly to perform complex biomarker electrochemical assays with excellent reproducibility. Additional effort, such as miniaturizing the readout system, however, is needed to fully realize this proof of concept as a portable handheld diagnostic, particularly in osteoporosis management.
